# The effect of task-irrelevant spatial contexts on 360-degree attention

**DOI:** 10.1371/journal.pone.0237717

**Published:** 2020-08-18

**Authors:** Yuki Harada, Junji Ohyama

**Affiliations:** 1 National Institute of Advanced Industrial Science and Technology, Human Augmentation Research Center, Tsukuba, Ibaraki, Japan; 2 Department of Rehabilitation for Brain Functions, Research Institute of National Rehabilitation Center for Persons with Disabilities, Tokorozawa, Saitama, Japan; University of Bath, UNITED KINGDOM

## Abstract

The effect of spatial contexts on attention is important for evaluating the risk of human errors and the accessibility of information in different situations. In traditional studies, this effect has been investigated using display-based and non-laboratory procedures. However, these two procedures are inadequate for measuring attention directed toward 360-degree environments and controlling exogeneous stimuli. In order to resolve these limitations, we used a virtual-reality-based procedure and investigated how spatial contexts of 360-degree environments influence attention. In the experiment, 20 students were asked to search for and report a target that was presented at any location in 360-degree virtual spaces as accurately and quickly as possible. Spatial contexts comprised a basic context (a grey and objectless space) and three specific contexts (a square grid floor, a cubic room, and an infinite floor). We found that response times for the task and eye movements were influenced by the spatial context of 360-degree surrounding spaces. In particular, although total viewing times for the contexts did not match the saliency maps, the differences in total viewing times between the basic and specific contexts did resemble the maps. These results suggest that attention comprises basic and context-dependent characteristics, and the latter are influenced by the saliency of 360-degree contexts even when the contexts are irrelevant to a task.

## Introduction

Attention plays a key role in detecting necessary information from numerous information sources [1]. Attentional characteristics are used to estimate the interaction between humans and environments, such as the risk of human error [2] and the accessibility of information [3]. For example, individual differences in attention act as one of the best predictors of traffic accidents [4]. In order to describe the underlying mechanisms of attention, numerous studies have investigated the characteristics of attention [5].

Many studies have found that spatial contexts influence the distribution and allocation of attention. For example, spatial attention is distributed in an elliptical manner around the fovea (useful field of view: UFOV [6]), which shrinks in various contexts such as the presence of distractors [7], emotional scenes [8], and recognition difficulty [9]. Similarly, the attentional allocation of eye movements is also biased by spatial contexts such as semantic inconsistency [10, 11], unexpectedness [12], negative emotion [13], and meaning [14]. Importantly, the saliency of spatial contexts has been addressed to estimate attentional allocation [15, 16]. Saliency is defined as a bottom-up uniqueness within a context such as color, orientation, shape, and other factors [17]. The saliency explanation has been supported by empirical evidence, especially for the early period of a scene inspection [18–20].

In order to measure the effect of spatial contexts on attention, previous studies have used two procedures: display-based and non-laboratory-based measurements. In the display-based measurement, participants report a target stimulus presented on a display [21, 22] or multiple displays [23]. However, this procedure is not adequate to measure attention directed toward 360-degree environments, as displays cannot fully cover participants’ surroundings. This is a limitation because individuals in real-life situations frequently shift their attention in 360-degree environments. On the other hand, in the non-laboratory-based measurement, participants perform a certain task in daily situations such as driving [24, 25] and making tea [26]. This procedure is effective for measuring attentional behaviours in 360-degree environments. However, it is difficult to control for exogeneous stimuli, such as auditory, visual, and other sensory information, preventing the separation of the effect of experimental factors from the effect of other unmanipulated factors.

In an attempt to resolve these two limitations, recent studies have used a new method using virtual reality (VR) technology [27–30]. In these studies, participants were asked to view or search certain scenes presented on virtual environments while their eye movements were recorded. The results showed several attentional characteristics. For example, the saliency of features influenced gaze patterns [27] and head movements [30]. These studies have suggested that 360-degree attention has context-dependent characteristics. On the other hand, basic characteristics in 360-degree attention was investigated by using a uniform visual environment without salient features (i.e., grey-colored background) [31]. The basic characteristic might be induced by endogenous factors in simple uniform contexts, and the context-dependent characteristic might be induced by exogenous factors of specific contexts such as saliency. Considering the two characteristics, it would be important to compare attentional behaviors between basic and specific contexts.

In this study, we compared attentional behaviors between basic and task-irrelevant specific contexts using the VR-based measurement. In the experiment, participants searched for and reported a target dot presented on a head-mounted display (HMD) as accurately and quickly as possible while their eye movements were recorded. In order to examine the effect of spatial context, we manipulated visual images of 360-degree virtual spaces (one basic and three specific contexts). If spatial contexts influence attention, the response times for the task and eye movements will be changed by the manipulation of the virtual spaces.

## Materials and methods

### Participants

Twenty students (9 men and 11 women) aged 20–27 years (*M* = 22.35, *SD* = 2.08) from the University of Tsukuba participated in the experiment. All participants had normal or corrected-to-normal visual acuity and were naïve as to the purpose of the experiment. The experiment was approved by the ethics committee of the National Institute of Advanced Industrial Science and Technology and conducted according to the principles of the Declaration of Helsinki. Written informed consent was obtained from all participants. The sample size was determined on the basis of Harada & Ohyama [31]. A post-hoc power test with G*Power showed that the power was .729. The value was comparable to the criteria (.8) introduced by Cohen [32].

### Apparatus and stimuli

Visual images were presented on an HMD using an application (Experiment Center 4.0, SensoMotoric Instruments). The HMD was equipped with an eye-tracking system (based on HTC Vive, SensoMotoric Instruments), with a sampling rate of 250 Hz. The presentation was controlled with a desktop PC (Alienware Area-51, Dell). Participants provided their responses using two wireless game pads (Dual shock 4, Sony Interactive Entertainment).

**Fig 1:**
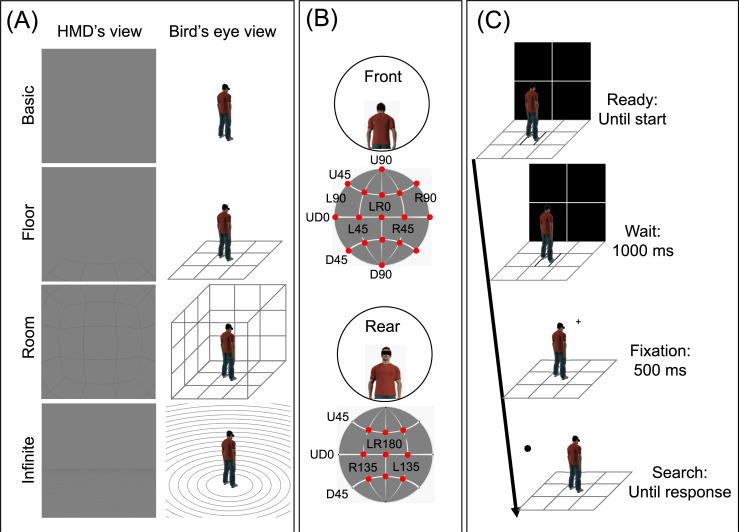
Schematic illustrations of experimental conditions. (A) The four spatial contexts. (B) The target location. Red dots show the target locations. (C) The sequence of a trial. The illustration shows an example of a trial sequence in the floor context.

As spatial contexts, we used four three-dimensional virtual images (Fig 1A): basic, floor, room, and infinite floor contexts. The basic context was a grey uniform 360-degree space without any objects. The floor context included a squared plane (90 × 90 degrees of visual angle) shown by dark grey grid lines. The room context was a cube composed of six planes drawn by dark grey grid lines surrounding a participant. The plane was identical to that in the floor context. The infinite floor was composed of numerous circled grids, perceived as a ground continuing to the horizon. In all the contexts, the RGB color value for the grey background was [128, 128, 128] and that for the dark gray lines was [115, 115, 115]. Additional 360-degree interactive figures are available here: https://sciadlab.wordpress.com/supplementary4plosone2020-vr-views.

As target stimuli for the search task, we used white ([R, G, B] = [255, 255, 255]) and black ([R, G, B] = [0, 0, 0]) dots, with a size of 1.5 × 1.5 degrees of visual angle. The target location was defined by a combination of latitude and longitude. The spatial interval between targets was 45 degrees, and the total number of target locations was 26 (Fig 1B).

### Procedure

The experiment lasted for 1.5 hours (rest times were included). The participants received instructions for the experiment and provided written informed consent. Subsequently, they wore the HMD and held one game pad in each hand.

The trial sequence was based on Harada & Ohyama [31] (Fig 1C). At the beginning of a trial, the participants oriented their bodies toward and fixated on the center of a white cross superimposed on a black square (90 × 90 degrees of visual angle). After the participants pressed the start button, the white cross remained for 1000 ms, and then a fixation cross (1.5 × 1.5 degrees of visual angle) was presented for 500 ms. Subsequently, a white or black target dot was presented at one of the 26 locations. In the target phase, the participants searched the 360-degree virtual space for the target dot and reported the color of the target as accurately and quickly as possible by pressing the game pad button. During the search, participants were allowed to move their body if needed. After the participants pressed the target button, the next trial began. Throughout the trials, the spatial context was constantly presented as a background stimulus.

The total number of trials was 416: spatial contexts (4) × target locations (26) × target color (2) × repetitions (2). The trials were conducted in eight experimental blocks. The spatial context was manipulated between blocks, and the order was counterbalanced across participants. The target locations were manipulated within blocks, and their order was randomized across blocks. The combination of target color and button was counterbalanced across participants.

### Analysis

In order to clarify the effect of spatial contexts on attention, we analyzed behavioral and eye movement data during the search phase. Behavioral data comprised correct response rate and time to complete the task. Eye movement data were analyzed in two ways. First, we analyzed total viewing times on each sub-area of the 360-degree virtual spaces. In this analysis, the 360-degree spaces were divided into 312 grid areas by imaginary 12 × 24 lines (spatial intervals were 15 degrees). The total viewing times were converted into heat maps using Surfer (Golden Software), with interpolations performed by the Kriging method [33]. Second, fixations, saccades, and coverage areas were coded irrespective of sub-areas. In the BeGaze algorithm (SensoMotoric Instruments), the threshold of saccade was defined as 40% of the maximum velocity in each trial, and fixation was defined as a gaze that dwelled for a minimal duration of 50 ms on a circular area spanning 2 degrees of visual angle in diameter. The coverage area is the 360-degree spatial area that participants visually searched. This area was calculated by assuming a 30-degree diameter around each fixation, although previous studies that used the display-based procedure assumed a 2–5-degree diameter [34, 35]. This is because the UFOV has been reported to cover a diameter of 30–60 degrees around each fixation in non-distractor conditions [3, 36]. Before this analysis, we computed the coverage area in each trial by assuming a 5-degree diameter as with the same procedure of a previous study [34] (S1 Fig).

In order to create saliency maps, we used the graph-based visual saliency technique [37]. This mapping was conducted using MATLAB and Image Processing Toolbox (Mathworks).

For statistical significance tests, analyses of variance (ANOVA) with the generalized-linear mixed-model were performed using the lmer function in the lmerTest package. We used a random intercept-model that included relevant factors and their interactions as fixed effects (random slopes were not entered because of model complexity limitations as in Bone et al. [38]). In this model, participants and/or target color were entered, as these variables were outside of the scope of this study. Multiple comparisons were performed using the lsmeans function in the lsmeans package, in which *p* values were adjusted with Tukey’s method. Regression analyses were conducted using the lm function in the default package. In order to clarify the difference between the spatial contexts, *p* values were converted into heat maps by using the geom_tile function in the ggplot2 package.

**Fig 2:**
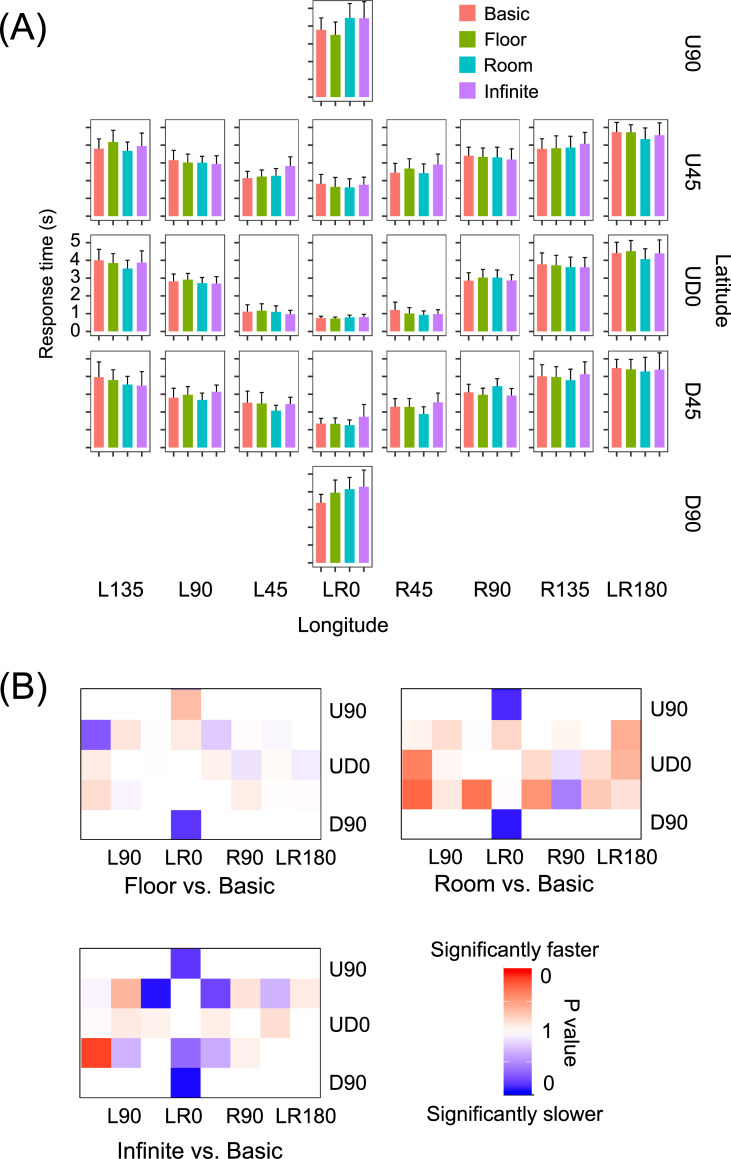
Effect of 360-degree spatial context on target detection. (A) Mean response times. Error bars represent 95% confidence intervals. (B) *P* values obtained from multiple comparisons.

In an attempt to reduce the effect of forward masking [39] due to the fixation mark, we excluded the data obtained from UD0 × LR0 and eye movement data obtained from immediately after the trial’s start (contained in a diameter of 7.5 degrees around the position of the fixation mark). Moreover, to reduce the effect of target detection in the peripheral vision, we excluded eye movements that were made immediately before target reports (within a diameter of 63.64 degrees around the target location). The diameter threshold was defined based on (a) response times, which significantly increased when the target was more than 63.64 degrees away from the fixation mark (see Fig 2A), and (b) previous reports, which showed that the size of the UFOV was approximately 30–60 degrees [3, 36]. In eye movement analyses, we excluded data obtained from eye blinking and outliers of saccade length (more than 180 degrees of visual angle).

## Results

### Correct response rate

Mean correct response rates were very high. Their 95% confidence intervals collapsed across the target locations were as follows: .978 to .985 for the basic context; .976 to .984 for the floor context; .968 to .979 for the room context: and .970 to .980 for the infinite floor context (see S2 Fig for data on each location). A two-way within-participants ANOVA was performed on correct response rate with the fixed effect of spatial context (4) and target locations (25). Participants and target colors were entered as random effects, including intercepts. The results showed a significant main effect of target locations [*F* (24, 455.8) = 1.651, *p* = .0281] and a non-significant main effect of the spatial context [*F* (3, 7424.5) = 1.371, *p* = .250]. The two-way interaction was not significant [*F* (72, 7424.5) = 0.966, *p* = .560]. Multiple comparison revealed that correct response rates were significantly lower at U45 × LR180 than at D45 × LR0 [*t* (456) = 3.816, *p* = .0318, *d* = 0.854]. All other differences were not significant [*t*s (456) < 3.562, *p*s > .0729, *d*s < 0.796].

### Response time

We analyzed response times (93.56% in all data) except for the data obtained from incorrect responses as well as extremely faster (less than 100 ms [40]) and slower response times (more than 2 *SD* from the mean [41]). Fig 2A shows the mean response times across the 20 participants. A two-way within-participants ANOVA was conducted on response times with the fixed effects of spatial context (4) and target locations (25). Participants and target colors were entered as random effects, including intercepts. The results showed a significant main effect of target location [*F* (24, 145.6) = 42.712, *p* < .0001] and a non-significant main effect of spatial context [*F* (3, 10.5) = 1.133, *p* = .380]. The two-way interaction was significant [*F* (72, 6858.2) = 1.416, *p* = .012]. The results of multiple comparisons are shown in Fig 2B. The differences in response times were significant for U90 [floor < infinite floor: *t* (662) = 3.287, *p* = .0059, *d* = 0.735; floor < room: *t* (678) = 3.457, *p* = .0033, *d* = 0.773, respectively], for U45 × L45 [infinite floor > basic: *t* (567) = 2.819, *p* = .0256, *d* = 0.630], for D45 × LR0 [room < infinite floor: *t* (586) = 2.663, *p* = .0396, *d* = 0.596], and for D90 [infinite floor > basic: *t* (768) = 3.070, *p* = .0119, *d* = 0.687; room > basic: *t* (715) = 2.683, *p* = .0374, *d* = 0.600, respectively]. All other differences were not significant (*t*s < 2.542, *p*s > .0547, *d*s < 0.568).

**Fig 3:**
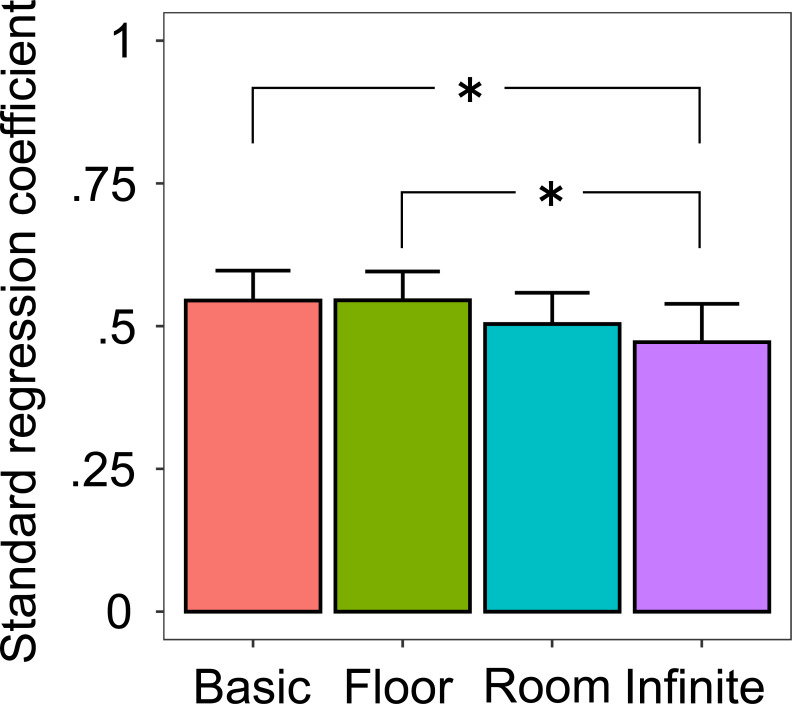
Effect of 360-degree spatial contexts on horizontal allocation of attention. Bars represent the mean standard regression coefficients as a function of target longitude. Error bars represent 95% confidence intervals. Asterisks represent significant differences (*p* < .05).

In order to examine the effect of spatial context on the horizontal allocation of attention, we analyzed the slope of response times as a function of target longitude. For each participant and spatial context, we conducted a regression analysis on response times with the parameter of the target longitude. Fig 3 shows the mean standard regression coefficients across 20 participants. A one-way within-participants ANOVA was performed on the standard regression coefficients with the fixed effect of spatial contexts (4). In this analysis, target color was not entered as a random effect because this factor was collapsed due to the regression analysis. Participants were entered as a random effect, including intercepts. The results showed a significant main effect [*F* (3, 57) = 4.836, *p* = .00456]. Multiple comparisons revealed that standard regression coefficients were significantly lower in the infinite floor context than in the basic and floor contexts [*t* (57) = 2.872, *p* = .0284, *d* = 0.642; *t* (57) = 3.069, *p* = .0168, *d* = 0.686, respectively]. The other differences were not significant [*t*s (57) < 2.431, *p*s > .0826, *d*s < 0.544]. The results of response times showed that spatial contexts influenced target detection. In particular, the infinite floor context facilitated the horizontal allocation of attention.

### Total viewing times

**Fig 4:**
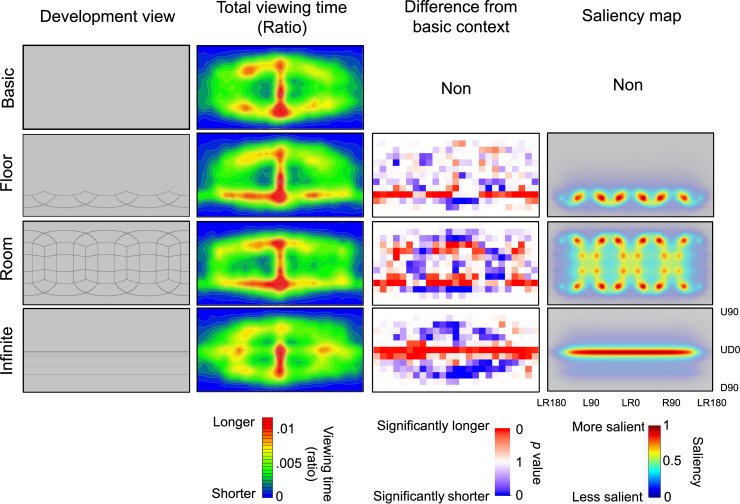
Effect of 360-degree spatial context on total viewing times. In the ratio of total viewing times, redder areas indicate longer viewing times, and more purple areas indicate shorter viewing times. The difference from the basic context shows *p* values obtained from multiple comparisons. The color becomes redder as the viewing time in a context was longer than in the basic context, while the color becomes bluer as the time was shorter than that in the basic context. The saliency map was created using the graph-based visual saliency technique.

In order to examine the effect of spatial contexts on attentional allocations, we computed the total viewing times for each of the 312 areas. Fig 4 shows the ratio of total viewing times averaged across the 20 participants. A two-way within-participants design ANOVA was performed on the ratio of total viewing times with the fixed effects of spatial contexts (4) and areas (312). Participants were entered as a random effect, including intercepts. In this analysis, target color was not entered as a random effect because target color was collapsed due to the calculation of total viewing time. The results revealed a significant main effect of area [*F* (311, 3166.9) = 13.90, *p* < .0001] but a non-significant main effect of spatial contexts [*F* (3, 18663.2) = 0, *p* = 1]. The results also revealed a significant interaction [*F* (933, 18663.2) = 3.396, *p* < .0001]. Fig 4 shows the results of multiple comparisons.

To examine the effect of saliency on attentional allocation, we created saliency maps of the four contexts (Fig 4). We found that the total viewing times were not matched to the saliency maps. However, the difference in total viewing times between the basic and non-basic contexts was considered to match the saliency maps.

### Saccades, fixations, and coverage

**Fig 5:**
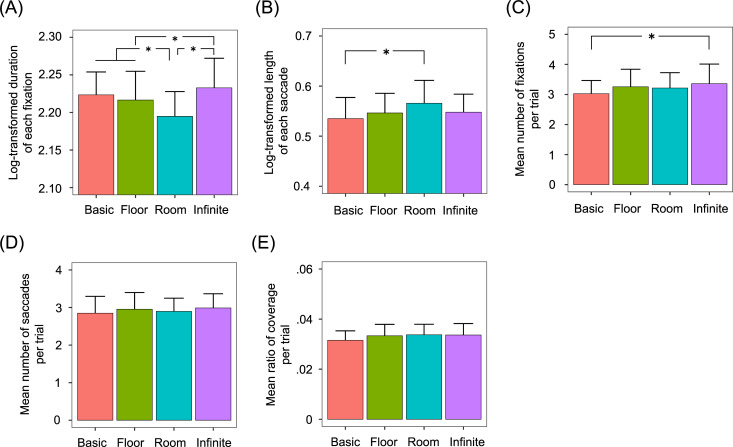
Effect of 360-degree spatial contexts on fixations and saccades. (A) Mean log-transformed duration of each fixation. (B) Mean log-transformed length of each saccade. (C) Mean number of fixations. (D) Mean number of saccades. All error bars represent 95% confidence intervals. Asterisks represent significant differences (*p* < .05).

For each participant and spatial context, we computed the duration of a fixation and the length of a saccade. Because the fixation duration and the saccade length did not follow the Gaussian function (*A* = 679.94, *p* < .0001; *A* = 4000.9, *p* < .0001, respectively), these were converted using log-transformation. Fig 5A, 5B, 5C and 5D show the duration of a fixation, the length of a saccade, and the number of fixations and saccades averaged across 20 participants. One-way within-participants ANOVAs were performed on the four variables with the fixed effect of spatial contexts (4). Participants, target locations, and target colors were entered as random effects because these variables were outside of the scope of this study. but can be considered to influence eye movements.

For the duration of a fixation, the main effect of spatial contexts was significant [*F* (3, 14592) = 14.717, *p* < .0001]. Multiple comparisons revealed that the fixation duration was significantly shorter in the room context than in the basic, floor, and infinite floor contexts [*t* (14595) = 4.105, *p* = .0002, *d* = 0.918; *t* (14595) = 3.006, *p* = .0141, *d* = 0.672; *t* (14582) = 6.53, *p* < .0001, *d* = 1.46, respectively]. The duration was also significantly longer in the infinite floor context than in the floor context [*t* (14596) = 3.503, *p* = .0026, *d* = 0.783]. The other differences were not significant (*t*s < 2.306, *p*s > .0968, *d*s < 0.516).

For the length of a saccade, the main effect of spatial contexts was significant [*F* (3, 21782) = 4.143, *p* = .00606]. A multiple comparison revealed that the saccade length was significantly larger in the room context than in the basic context [*t* (21797) = 3.484, *p* = .0028, *d* = 0.779]. All other differences were not significant (*t*s < 2.172, *p*s > .131, *d*s < 0.486).

For the number of fixations, the main effect of spatial contexts was significant [*F* (3, 7452.1) = 4.839, *p* = .00230]. Multiple comparisons revealed that the number of fixations was significantly larger in the infinite context than in the basic context [*t* (7452) = 3.738, *p* = .0011, *d* = 0.856]. The other differences were not significant (*t*s < 2.502, *p*s > .0597, *d*s < 0.560). For the number of saccades, the main effect was not significant [*F* (3, 7452.1) = 0.765, *p* = .514].

For each participant and spatial context, we calculated the ratio of coverage. Fig 5E shows the coverage ratio in each trial averaged across 20 participants. A one-way within-participants ANOVA was performed on the coverage ratio with the fixed effect of spatial contexts (4). For the same reason as in the fixation and saccade analyses, participants, target locations, and target colors were entered as random effects. The results revealed that the main effect of spatial contexts was not significant [*F* (3, 7452.1) = 2.523, *p* = .0559].

## Discussion

In this experiment, we investigated how spatial contexts of 360-degree environments influence attention. We found that spatial contexts influenced response times in the search task and eye movements made in 360-degree virtual spaces. Our findings provide evidence that attention is biased by task-irrelevant spatial contexts even in 360-degree environments.

Response time data showed that spatial contexts of 360-degree environments influence the speed of target detection. In particular, the infinite floor context advanced the horizontal allocation of attention. A reason for these results may be related to the depression of attentional allocation between different-depth planes [42, 43]. In the infinite floor context, perceived depth dynamically changes in the vertical direction but does not change in the horizontal direction. The differences in perceived depth in the vertical direction would facilitate the horizontal allocation of attention.

Regarding total viewing times, we obtained two important results. First, in the basic context, attention spread from the front of participants toward latitudinal and longitudinal directions. Because this is consistent with previous results [27, 32], this basic distribution is considered to be robust. Second, although the distributions of total viewing times were not similar to the saliency maps, the difference in total viewing times between the basic and other contexts was similar to the saliency maps. These results suggest that 360-degree attention comprises basic and context-dependent characteristics. This hypothesis assumes that attention in a specific context is explained by combining a basic distribution of attention with a context-dependent distribution such as saliency. This hypothesis is indirectly supported by previous works, in which basic attention was distributed in an elliptical manner [3, 36] but biased by contexts [21]. Unfortunately, because only small studies have measured the basic and context-dependent distributions of attention in 360-degree environments, the extent to which this hypothesis explains 360-degree attention has remained unclear. Therefore, future works will be required to examine this issue.

The results also showed that the total viewing time was longer on salient features such as the edge and boundary of the floor context, the side of the room context, and the horizon of the infinite floor context than on other areas, even when the spatial contexts were irrelevant to the task. The effect of saliency on attention is also supported by the fixation duration and the saccade length. For example, the room context produced a larger length of saccade and a smaller duration of fixation, perhaps related to the fact that the room context had many salient areas in omni-directions. In this context, attention shifts toward various areas of the 360-degree environment, resulting in larger saccade length and shorter fixation duration. These results are consistent with previous research on human attention and cognitive neuroscience [44]. Neuroscience studies have suggested that visual features such as edges, boundaries, and object shape are informative for recognizing three-dimensional structures of space and objects [45, 46]. Our results imply that edges and boundaries were automatically detected for recognizing the three-dimensional structure of a space even though the spatial context was task-irrelevant. This unconscious process requires the effective detection of salient features, which affects attentional allocation in 360-degree environments.

A potential limitation of our analysis is the difference in the data structures between the total viewing times and saliency maps. The saliency maps were created by 2D images that were projected by an equirectangular method, although the total viewing times were obtained from actual viewing conditions composed of a 3D closed sphere structure. Because the calculation of a saliency map for a 3D closed celestial sphere structure is not standardized, it is difficult to compare these different dimensional data quantitatively. The resolution for this issue requires future analyses that calculate the saliency in a 3D closed sphere structure.

The present data suggest that the saliency of objects impairs the accessibility of other necessary information in 360-degree environments. In such a situation, certain techniques would be useful to guide users’ attention towards certain locations. For example, Veas et al. [47] developed a technique to modify the saliency of objects by using augmented reality (AR). This technique can guide users’ attention towards necessary objects with small cognitive loads. Another possibly effective technique is visual guidance, in which the location of necessary objects is depicted with specific designs such as an arrow and a three-dimensional radar [48, 49]. This technique can strongly guide attention towards necessary objects [50] but involves a small cognitive load. These techniques would be useful for enhancing accessibility in various 360-degree environments.

## Conclusions

We found that spatial contexts of 360-degree virtual spaces influenced response times for target search and eye movements. Importantly, the differences in total viewing times between the basic and other contexts were similar to the saliency maps. These results suggest that attention comprises basic and context-dependent characteristics, and the latter is influenced by the saliency of 360-degree contexts even when the contexts are irrelevant to a task.

## Supporting information

S1 Fig Effect of spatial contexts on the coverage area. The coverage area was computed by assuming a 5-degree diameter in each fixation. Bars represent means, and error bars represent 95% confidence intervals.
(EPS)

S2 Fig Effect of spatial contexts on the target detection. Point plots represent the means, and error bars represent 95% confidence intervals. Grey dots represent the correct response rate of each participant.
(EPS)

S1 Data Data for figures. The data required to create figures were shown in this file.
(XLSX)
